# The effects of sequential therapy using anti-resorptive agents after administering once-weekly teriparatide or twice-weekly teriparatide

**DOI:** 10.1007/s00774-026-01690-7

**Published:** 2026-01-31

**Authors:** Hidehiro Matsumoto, Manabu Tsukamoto, Nobukazu Okimoto, Satoshi Ikeda, Masahiro Tanaka, Mitsugu Takahashi, Yoshiaki Ikejiri, Fumihiro Oha, Satoshi Mizuno, Keiichi Shigenobu, Akinori Sakai, Junichi Takada

**Affiliations:** 1 Department of Orthopaedic Surgery, Sanzai Hospital, Saito, Japan; 2 Department of Orthopaedic Surgery, University of Occupational and Environmental Health, 1-1 Iseigaoka, Yahatanishi-Ku, Kitakyushu, Japan; 3 Okimoto Clinic, Kure, Japan; 4 Department of Orthopaedic Surgery, Ken-Ai Memorial Hospital, Onga, Japan; 5 Department of Orthopaedic Surgery, MAZDA Hospital, Fuchu, Japan; 6 Takahashi Orthopedics Clinic, Chitose, Japan; 7 Department of Orthopaedic Surgery, Shimura Hospital, Hiroshima, Japan; 8 Department of Orthopaedic Surgery, Hakodate Central General Hospital, Hakodate, Japan; 9 Hanakawa Orthopedics Clinic, Sapporo, Japan; 10 Haraya Orthopedics Clinic, Sapporo, Japan; 11 Osteoporosis Center, Sapporo Maruyama Orthopaedic Hospital, Sapporo, Japan

**Keywords:** Teriparatide, Bone structure, Bone strength, 3D modeling, 3D-DXA

## Abstract

**Introduction:**

Studies launched in Japan on once-weekly teriparatide (1/W-TPTD) and twice-weekly teriparatide (2/W-TPTD) are limited. Therefore, we examined the effects of sequential therapy using anti-resorptive agents after administering 1/W-TPTD and 2/W-TPTD using dual-energy X-ray absorptiometry (DXA) and DXA-based 3D modeling.

**Materials and methods:**

This was a multicenter retrospective study following a phase 3 clinical trial called the TWICE study. Two-year follow-up data were collected after administering 1/W-TPTD or 2/W-TPTD for 1 year (follow-up after the phase 3 clinical trial).

**Results:**

20 subjects in the group of pre-treatments with 1/W-TPTD followed by sequential administration of bisphosphonate or denosumab (1/W-TPTD [BP/denosumab]), and 22 in the group 2/W-TPTD (BP/denosumab) were included in the analysis of changes in the BMD by post-treatment. In the 1/W-TPTD (BP/denosumab) group, a significant increase in L2–4 BMD was observed. In the 2/W-TPTD (BP/denosumab) group, a significant increase in total hip, neck, and L2–4 BMD values was observed. Analysis by 3D-SHAPER revealed that both the 1/W-TPTD (BP/denosumab) and 2/W-TPTD (BP/denosumab) groups demonstrated significant increases in cortical sBMD and vBMD 2 years after the initiation of post-treatment.

**Conclusion:**

In subjects who received 1/W-TPTD and 2/W-TPTD for about 1 year followed by sequential administration of BP or denosumab, significant improvements in BMD were continuously observed. Furthermore, significant improvements in cortical sBMD and vBMD were also demonstrated by analysis using 3D-SHAPER. Both 1/W-TPTD and 2/W-TPTD were effective in the treatment of osteoporosis by using anti-resorptive agents for sequential administration.

**Supplementary Information:**

The online version contains supplementary material available at 10.1007/s00774-026-01690-7.

## Introduction

In severe osteoporosis with a high risk of bone fractures, it is recommended to initiate the administration of teriparatide (TPTD) and romosozumab, which facilitate osteogenesis with anabolism, or denosumab (denosumab) that suppresses bone resorption earlier [1, 2]. TPTD has been reported to increase bone mineral density (BMD) and improve bone quality [3]. However, due to concerns about the risk of bone tumors [4, 5], the duration of treatment with TPTD is still limited to a maximum of 2 years in Japan. TPTD must be switched to another medication if patients have completed receiving it because of the concern that BMD would decrease after terminating TPTD treatment [6, 7].

It has been previously reported that sequential administration of an anti-resorptive agent, such as denosumab and bisphosphonate (BP), can be effective in maintaining and increasing BMD after the completion of TPTD administration [8–10], and that BMD in the lumbar spine may further be increased by maintenance therapy with denosumab rather than BP [11]. On the other hand, it has also been reported that the decrease in BMD was not able to be suppressed by treatment with raloxifene, a selective estrogen receptor modulator (SERM) [12].

All of those results are mainly based on reports on daily TPTD (DAILY TPTD), and the number of studies for once-weekly teriparatide (1/W-TPTD) and twice-weekly teriparatide (2/W-TPTD) that have been launched in Japan is limited. Nakamura et al. reported that 1/W-TPTD administration reduced the cumulative incidence of new vertebral fractures in patients with osteoporosis at higher fracture risk [13], and Sugimoto et al. reported that 2/W-TPTD can provide comparable efficacy to 1/W-TPTD while improving safety [14]. However, there have been no reports of sequential administration in subjects who received 1/W-TPTD and 2/W-TPTD, and we hypothesized that sequential therapy using anti-resorptive agents after administration of 1/W-TPTD or 2/W-TPTD would further increase BMD at each site.

For the above reasons, we retrospectively conducted a follow-up study after the completion of the multicenter, randomized, double-blind, double-dummy, active-controlled, non-inferiority trial (TWICE study [14]) to evaluate the effects of sequential therapy after 2/W-TPTD or 1/W-TPTD.

## Materials and methods

### Study design

In the present study, prognoses were examined only in subjects undergoing an examination by the same manufacturer’s device among those who participated in the 48-week clinical trial. Data obtained from the study for 48 weeks from the start of study treatment were examined in accordance with the Declaration of Helsinki, Good Clinical Practice (GCP), and the protocol approved by the institutional review board at each site. Prognosis survey data over 2 years subsequent to the study were used for a retrospective study in accordance with the Declaration of Helsinki, ethical guidelines by the Ministry of Health, Labour and Welfare, and the protocol approved by the ethics review committee at the Tomonaga Clinic (date of approval by the ethics review committee, October 7, 2021; Approval No. 20211007).

Among the study data, those for the first 48 weeks were collected by the following prospective intervention study [14].The TWICE study involved 553 subjects who participated in a 48-week, multicenter, randomized, double-blind, double-dummy, active-controlled, non-inferiority study conducted in Japan and who were randomized to a group receiving 28.2 µg TPTD twice weekly and placebo once weekly or a group receiving 56.5 µg TPTD once weekly and placebo twice weekly in a 1:1 ratio by dynamic allocation based on the minimization method. The injections of 28.2 μg TPTD twice weekly and placebo were to be self-administered with an autoinjector by the subjects, in principle, every 3 or 4 days (with 2 or 3 days between injection days). The injections of 56.5 μg TPTD once weekly and placebo were administered by a healthcare professional during outpatient visits. The subjects also received concomitant treatment with daily oral calcium 610 mg, vitamin D3 400 IU, and magnesium 30 mg during the TWICE study (Shin Calcichew D3, Takeda Consumer Healthcare Company Limited, Osaka, Japan). At approximately 3 years after the end of the study, we decided to perform a retrospective survey and obtained approval from the ethics review committee for data accumulation. The sequential medications, for which data were collected retrospectively, were determined at the discretion of a physician specializing in osteoporosis.

### Study subjects

Inclusion criteria (the follow-up study).

Patients who met the following inclusion criteria were considered eligible for the study:

- Patients enrolled in the TWICE study from January to July 2017 and who received 1/W-TPTD or 2/W-TPTD for 48 weeks.
- Patients who underwent DXA measurements using the device from the same manufacturer (Hologic, Inc.).
- Patients who visited the study site after the end of the clinical trial and for whom follow-up was available.

Inclusion criteria at the start of the clinical trial

At the start of the clinical trial, patients considered to be at very high risk of fractures and who participated in the study met all of the following criteria:

- A diagnosis of primary osteoporosis according to the 2012 revision of the diagnostic criteria for primary osteoporosis. Patients having no diseases other than osteoporosis that cause low bone mass or secondary osteoporosis, and the results of their bone assessment meeting the following criteria: if fragility fractures are present, 1) a vertebral fracture or proximal femoral fracture is observed, or 2) other fragility fractures are present and BMD is less than 80% of young adult mean (YAM). If no fragility fractures are present, BMD is less than 70% of YAM or less than -2.5SD [15].
- Aged 65 years or older.
- A history of fractures of at least one to five bones, including those in the region between the fourth thoracic spine (Th4) and the lumbar spine (L4).
- A baseline BMD in the lumbar spine (L2–L4) that was less than 80% of YAM.
- Outpatients who were able to walk independently.
- Patients who were capable of self-administering the medication.

Exclusion criteria (the follow-up study).

Patients were excluded from the study if they refused to use their data due to opting out, or if they were deemed unsuitable for the study by the investigator or researcher.

The exclusion criteria at the start of the clinical trial.

At the start of the clinical trial, patients who participated in the study met none of the following criteria.

- A diagnosis of secondary osteoporosis.
- A disease accompanied by bone loss other than osteoporosis.
- An X-ray finding that might affect the BMD in the lumbar spine measured by DXA.
- A serum calcium level of 11.0 mg/dL or higher.
- A malignant or metastatic bone tumor.
- A history of radiotherapy that may affect the bones, or other factors increasing the risk of osteosarcoma.
- A serum alkaline phosphatase level greater than twice the reference value.
- A history of treatment with TPTD and/or anti-receptor activator of nuclear factor κB ligand antibody.
- Recent treatment with bisphosphonate (within the last 52 weeks).
- Recent treatment with other osteoporosis medications (within the last 8 weeks).
- Other factors determined by the investigator or researcher to be inappropriate for the study.

### Evaluation

The primary end point was the percentage changes in the BMD of the lumbar spines (L2–4) from the start of post-treatment to 2 years after starting post-treatment. Secondary end points included total hip, femoral neck (neck), and L2–4 BMD values from the start of post-treatment to 1 and 2 years after starting post-treatment, percentage changes in the analytic value of the femoral cortical bone measured by 3D-SHAPER*, percentage changes in the BMD and analytic value of the femoral cortical bone measured by 3D-SHAPER from the start of pre-treatment to a year after starting pre-treatment (the start of post-treatment), and 1 and 2 years after starting post-treatment. These were analyzed for within-groups variation. In the analyses by 3D-SHAPER, the changes at 2 years after starting post-treatment were visualized three dimensionally.

*A software application for analyses similar to QCT, brief evaluation of the three-dimensional (3D) bone structure is currently available by creating a unique 3D model of a patient with a 3D statistical model. It is obtained from two-dimensional (2D) images of the femoral bone taken by the DXA method, which requires no exposure to X-rays and additional examinations [16].

Subjects who had at least one BMD data point at 1 or 2 years after starting post-treatment were included in the analysis for the efficacy of the post-treatment. Four subgroups for the efficacy evaluation included the following: [A] pre-treatment with 1/W-TPTD, followed by sequential administration of BP or denosumab (1/W-TPTD [BP/denosumab]), [B] pre-treatment with 1/W-TPTD followed by post-treatment with SERM for sequential administration or without sequential administration (1/W-TPTD [others]), [C] pre-treatment with 2/W-TPTD followed by sequential administration of BP or denosumab (2/W-TPTD [BP/denosumab]), and [D] pre-treatment with 2/W-TPTD followed by post-treatment with SERM for sequential administration or without sequential administration (2/W-TPTD [others]). Regarding the handling drugs for post-treatment, when there were other drugs for sequential administration, the drugs that was used for at least 6 months after the clinical trial were handled as the first drugs by follow-up after a year, and their subsequent data were excluded. When there were no other drugs for sequential administration, the drugs that was used during at least two-thirds of a period by the end of follow-up (FU) at 2 years after the clinical trial (Supplement 1). Data at 48 weeks (about a year) after starting pre-treatment were used as those at the start of post-treatment. DXA-based 3D modeling analysis (3D-SHAPER, version 2.10.1; 3D-SHAPER Medical SL, Barcelona, Spain) was used for structural analysis of the proximal femur.

### Statistical analysis

For BMDs and 3D-shaped analysis data, changes over time within each group were assessed by the paired *t*-test. Patient characteristics were compared using Student’s *t*-test for continuous variables and Fisher’s exact test for nominal variables. In this study, to evaluate the effects of sequential administration, the analyses were conducted in four subgroups: 1/W-TPTD (BP/denosumab), 1/W-TPTD (others), 2/W-TPTD (BP/denosumab), and 2/W-TPTD (others). Statistical analyses were performed using R version 4.1.2 (R Foundation for Statistical Computing, Vienna, Austria [https://www.R-project.org/]). All statistical tests were performed with a significance level of 0.05. Data are presented as means ± standard error (SE) or numbers (proportion) of patients.

## Results

**Fig. 1:**
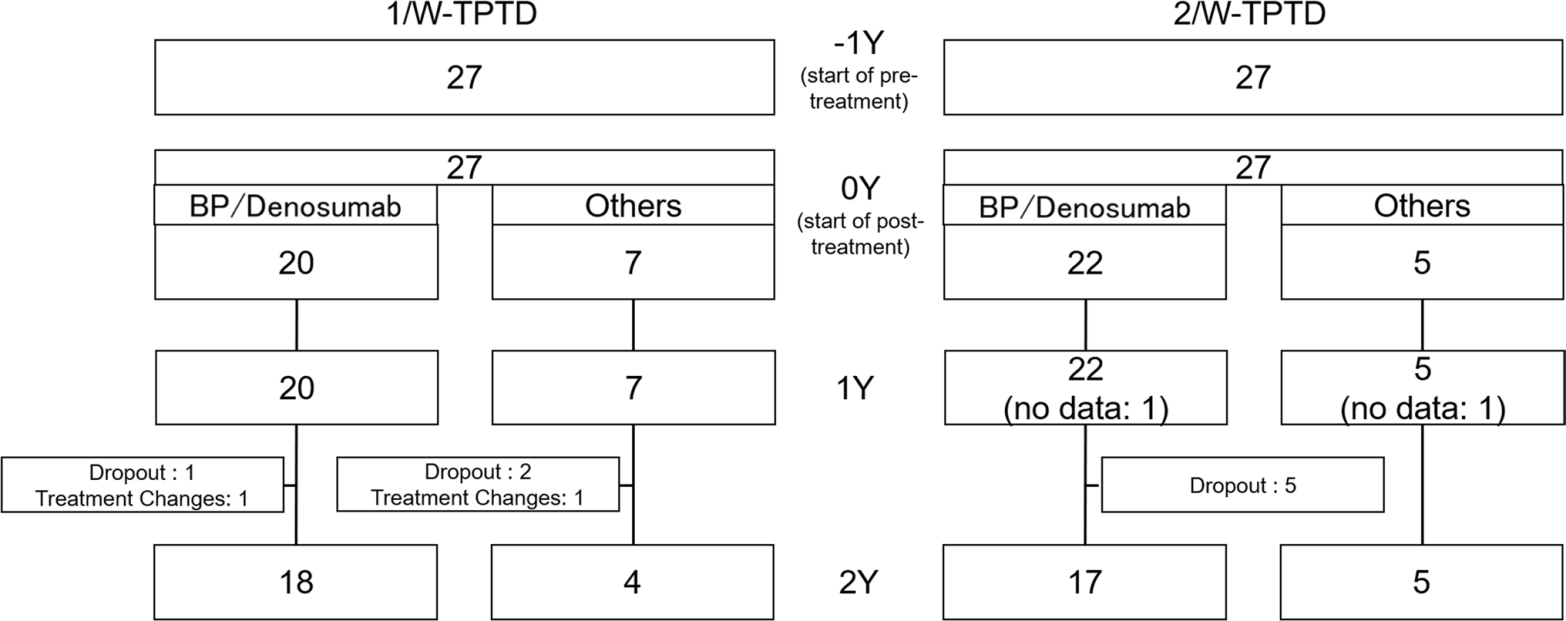
Participant flow. For DXA, the number of subjects with L2–4 BMD is indicated (the number of subjects with L2–4 BMD that was largest in DXA was selected). Some subjects had only data on the markers and safety evaluation without DXA measurements. Pre-treatment period: duration of the clinical trial in the prospective study; post-treatment period: follow-up period after the clinical trial (the post hoc retrospective study); 2/W TPTD: twice-weekly teriparatide; 1/W TPTD: once-weekly teriparatide; BP/denosumab: bisphosphonate or denosumab

Five subjects who had no follow-up efficacy analysis data, one subject who changed sequentially administered medication within a short period of time, and two subjects treated with 1/W-TPTD for post-treatment were excluded from the efficacy analysis for post-treatment. Follow-up data after the clinical trial could be collected from 27 subjects in the 1/W-TPTD group and 27 in the group 2/W-TPTD (Fig. 1). Among those subjects, 20 in the 1/W-TPTD (BP/denosumab) group, 7 in the 1/W-TPTD (others) group, 22 in the 2/W-TPTD (BP/denosumab) group, and 5 in the group 2/W-TPTD (others) were included in the analysis of changes in the BMD by post-treatment. Patient characteristics are shown in Table 1. For reference, we also provide patient characteristics on all cases that switched to BP/denosumab and those who switched to others in Supplement 2.

**Table 1:** Patient characteristics

	1/W-TPTD (BP/denosumab)	1/W-TPTD (others)	*p*	2/W-TPTD (BP/denosumab)	2/W-TPTD (others)	*p*	
*n*	20	7		22	5		
Age (years)	73.4 ± 6.0	77.9 ± 1.9	0.088	75.5 ± 1.2	75.0 ± 2.3	0.857	a
Height (cm)	149.9 ± 6.3	151.4 ± 2.9	0.630	150.1 ± 1.5	150.7 ± 1.3	0.846	a
Weight (kg)	52.5 ± 6.3	53.7 ± 2.7	0.691	49.0 ± 1.4	54.2 ± 4.0	0.146	a
Body mass index (kg/m^2^)	23.5 ± 3.7	23.6 ± 1.7	0.959	21.9 ± 0.8	23.9 ± 1.9	0.304	a
25-OH vitamin D3 (ng/mL)	27.6 ± 8.4	25.6 ± 1.6	0.550	24.2 ± 1.1	26.2 ± 4.3	0.537	a
Estimated GFR (mL/min/1.73m^2^)	68.6 ± 14	65.6 ± 5.2	0.623	62.7 ± 3.1	64.8 ± 7.6	0.781	a
Complications							
Type 2 diabetes mellitus	1 (5%)	0 (0%)	1.000	1 (4.5%)	1 (20%)	0.342	b
Chronic kidney disease (CKD)	1 (5%)	1 (14.3%)	0.459	2 (9.1%)	0 (0%)	1.000	b
Hypertension	2 (10%)	2 (28.6%)	0.269	4 (18.2%)	1 (20%)	1.000	b
Dyslipidemia	2 (10%)	1 (14.3%)	1.000	2 (9.1%)	1 (20%)	0.474	b
Knee osteoarthritis	1 (5%)	2 (28.6%)	0.156	3 (13.6%)	2 (40%)	0.144	b
Sex							
Male	1 (5%)	0 (0%)	1.000	4 (18.2%)	0 (0%)	0.561	a
Female	19 (95%)	7 (100%)		18 (81.8%)	5 (100%)		
Postmenopausal duration (years)							
10–< 20	8 (40%)	0 (0%)	0.080	2 (9.1%)	0 (0%)	0.703	b
≥ 20	11 (55%)	7 (100%)		16 (72.7%)	5 (100%)		
Male	1 (5%)	0 (0%)		4 (18.2%)	0 (0%)		
Non-vertebral fractures without large external force occurring at or after age 50 years							
Yes	5 (25%)	2 (28.6%)	1.000	10 (45.5%)	2 (40%)	1.000	b
No	15 (75%)	5 (71.4%)		12 (54.5%)	3 (60%)		
Medical history relevant to bone metabolism							
Yes	1 (5%)	2 (28.6%)	0.156	4 (18.2%)	0 (0%)	0.561	b
No	19 (95%)	5 (71.4%)		18 (81.8%)	5 (100%)		
Current smoking							
Yes	0 (0%)	0 (0%)	NA	1 (4.5%)	0 (0%)	1.000	b
No	20 (100%)	7 (100%)		21 (95.5%)	5 (100%)		
Alcohol consumption (3 or more units/day)							
Yes	0 (0%)	0 (0%)	NA	0 (0%)	0 (0%)	NA	b
No	20 (100%)	7 (100%)		22 (100%)	5 (100%)		
Parent fractured hip							
Yes	3 (15%)	1 (14.3%)	1.000	5 (22.7%)	0 (0%)	0.547	b
No	17 (85%)	6 (85.7%)		17 (77.3%)	5 (100%)		
Prior medications for osteoporosis							
Yes	11 (55%)	4 (57.1%)	1.000	11 (50%)	4 (80%)	0.342	b
No	9 (45%)	3 (42.9%)		11 (50%)	1 (20%)		
Number of vertebral fractures at baseline							
0	5 (25%)	2 (28.6%)	0.633	5 (22.7%)	2 (40%)	0.666	b
1	12 (60%)	3 (42.9%)		12 (54.5%)	3 (60%)		
2–3	2 (10%)	2 (28.6%)		5 (22.7%)	0 (0%)		
4–5	0 (0%)	0 (0%)		0 (0%)	0 (0%)		
Missing or not reported	1 (5%)	0 (0%)		0 (0%)	0 (0%)		
Lumbar spine BMD (based on YAM) (L2–L4) (%)	67.9 ± 7.3	68.7 ± 2.7	0.788	66.1 ± 2.2	73.0 ± 4.2	0.177	a
Lumbar spine BMD (based on YAM) (L1–L4) (%)	68.8 ± 7.8	69.3 ± 2.8	0.885	66.4 ± 2.0	72.9 ± 3.6	0.174	a
Femoral neck BMD (based on YAM) (%)	65.0 ± 8.2	66.4 ± 3.3	0.089	66.0 ± 2.6	69.8 ± 4.0	0.188	a
Total hip BMD (based on YAM) (%)	71.0 ± 7.5	76.6 ± 2.4	0.699	74.0 ± 2.5	81.6 ± 4.7	0.513	a
Sequential administration							
1/W TPTD	0 (0%)	0 (0%)		0 (0%)	0 (0%)		
BP	14 (70%)	0 (0%)		17 (77.3%)	0 (0%)		
D-mab	6 (30%)	0 (0%)		5 (22.7%)	0 (0%)		
SERM	0 (0%)	2 (28.6%)		0 (0%)	2 (40%)		
Nothing	0 (0%)	5 (71.4%)		0 (0%)	3 (60%)		
BP/D-mab	20 (100%)	0 (0%)		22 (100%)	0 (0%)		
Others (SERM/nothing)	0 (0%)	7 (100%)		0 (0%)	5 (100%)		
BP							
ALN	4 (28.6%)			6 (35.3%)			
RIS	3 (21.4%)			3 (17.6%)			
MIN	2 (14.3%)			5 (29.4%)			
IBN	4 (28.6%)			3 (17.6%)			
ZOL	1 (7.1%)			0 (0%)			
Concomitant drug after the clinical trial							
Active vitamin D3 preparation	15 (75%)	2 (28.6%)	0.065	10 (45.5%)	2 (40%)	1.000	b
Natural vitamin D3 preparation	1 (5%)	0 (0%)	1.000	1 (4.5%)	0 (0%)	1.000	b
Calcium preparation	2 (10%)	0 (0%)	1.000	1 (4.5%)	0 (0%)	1.000	b

Data are n (%) or mean ± standard deviation

^a^Student's *t* test; ^b^Fisher’s exact test

*GFR* glomerular filtration rate, *BMD* bone mineral density, *YAM* young adult mean, *1/W-TPTD* once-weekly teriparatide, *2/W-TPTD* twice-weekly teriparatide, *BP* bisphosphonate, *D-mab* denosumab, *SERM* selective estrogen receptor modulator, *ALN* alendronate, *RIS* risedronate, *MIN* minodronic acid, *IBN* ibandronate, *ZOL* zoledronic acid

### Changes in the BMD at a year and 2 years after starting post-treatment

**Fig. 2:**
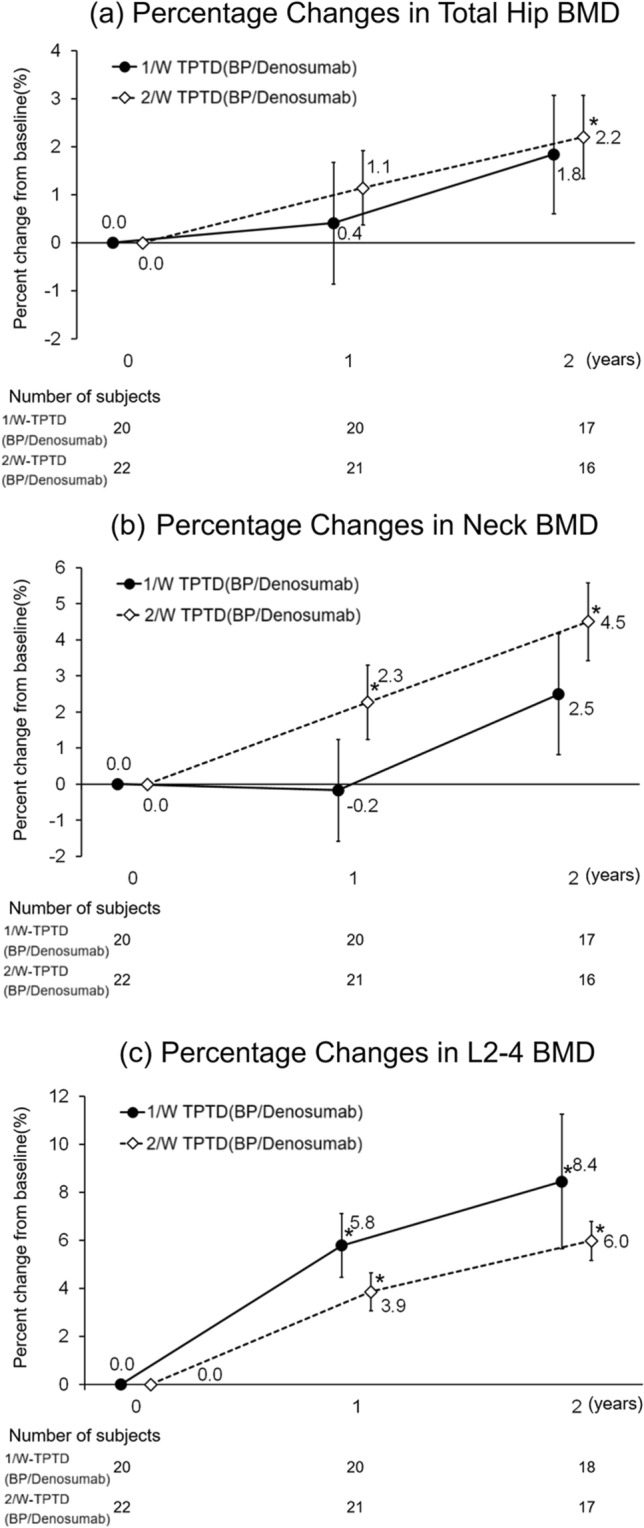
Changes in the BMD at 1 year and 2 years after starting post-treatment. *p < 0.05 versus 0-year, paired *t* test. Data are mean ± standard error. *BMD* bone mineral density; *2/W TPTD* twice-weekly teriparatide; *1/W TPTD* once-weekly teriparatide

In the 1/W-TPTD (BP/denosumab) group, a significant increase in L2–4 BMD was observed; the percentage changes were + 5.8% at a year after starting post-treatment and + 8.4% at 2 years after starting post-treatment on a continuous basis (Fig. 2). In the 2/W-TPTD (BP/denosumab) group, a significant increase in total hip BMD values was observed; the percentage changes were + 2.2% at 2 years after starting post-treatment. A significant increase in neck and L2–4 BMD values was observed at both a year and 2 years after starting post-treatment (neck, + 2.3% and + 4.5%, respectively; L2–4, + 3.9% and + 6.0%, respectively; Fig. 2). No significant changes were observed in the 1/W-TPTD (others) and 2/W-TPTD (others) groups. (Supplement 3) In addition, there were several patients who only underwent L2–4 examinations and did not undergo total hip and neck examinations.

### Changes in BMD from the start of pre-treatment to 2 years after starting post-treatment

In the 1/W-TPTD (BP/denosumab) group, significant increases in L2–4 BMD on a continuous basis were observed. Total hip, neck BMD values were unchanged from the start of pre-treatment, as shown in Supplement 4. No significant changes were observed in the 1/W-TPTD (others) group, as shown in Supplement 5.

In the 2/W-TPTD (BP/denosumab) group, significant increases in L2–4 BMD on a continuous basis were observed in the group for which the treatment was switched to BP/denosumab. Total hip and neck BMD values were also continuously increased after starting pre-treatment, and significant increases in total hip and neck BMD values were observed at 2 years, as shown in Supplement 4. In the 2/W-TPTD (others) group, even with a significant increase in L2–4 BMD at the start of post-treatment, no significant changes were observed afterward compared to the start of pre-treatment, as shown in Supplement 5.

### Changes in femoral bone parameters of 3D-SHAPER at 2 years after starting post-treatment

**Fig. 3.**
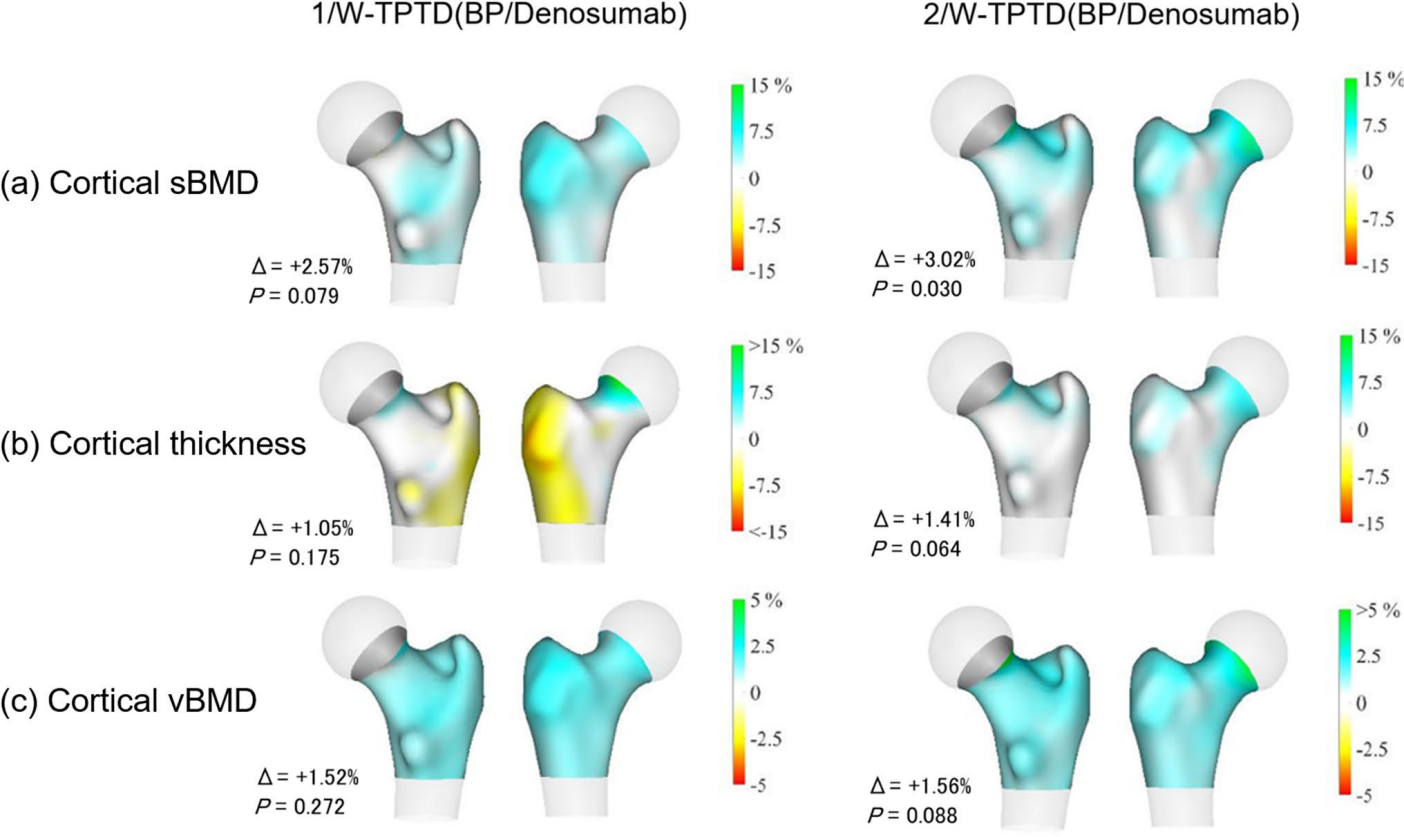
3D visualizations showing percentage changes from start of post-treatment to 2 years after starting post-treatment in cortical sBMD (**a**), cortical thickness (**b**), and cortical vBMD (**c**). There were 15 cases in each group. *BMD* bone mineral density; *1/W TPTD* once-weekly teriparatide; *2/W TPTD* twice-weekly teriparatide.

The numbers of subjects from whom all analysis results by 3D-SHAPER were obtained at three time points, including the start of pre-treatment, the start of post-treatment, and 2 years after starting post-treatment, were 15 in the 1/W-TPTD (BP/denosumab) group, 4 in the 1/W-TPTD (others) group, 15 in the 2/W-TPTD group (BP/denosumab), and 5 in the 2/W-TPTD (others) group. Among those subjects, cross sections by which changes in anatomical distributions of cortical bone structure (cortical sBMD, cortical thickness, and cortical vBMD) at 2 years after starting post-treatment were visualized three dimensionally were in the 1/W-TPTD (BP/denosumab) and 2/W-TPTD (BP/denosumab) groups [Fig. 3 shows data of the groups 1/W-TPTD (BP/denosumab) and 2/W-TPTD (BP/denosumab), and Supplement 6 shows data of the groups 1/W-TPTD (others) and 2/W-TPTD (others)]. The colors blue/green and yellow/red mean “increase” and “decrease,” respectively. In the 1/W-TPTD (BP/denosumab) and 2/W-TPTD (BP/denosumab) groups, significant increases in cortical sBMD and cortical vBMD were observed at 2 years after starting post-treatment. Moreover, the cross sections showing changes in cortical and trabecular vBMD at 2 years after starting post-treatment are indicated in Supplement 7.

### Analysis results by 3D-SHAPER from the start of pre-treatment to 2 years after starting post-treatment

**Fig. 4.**
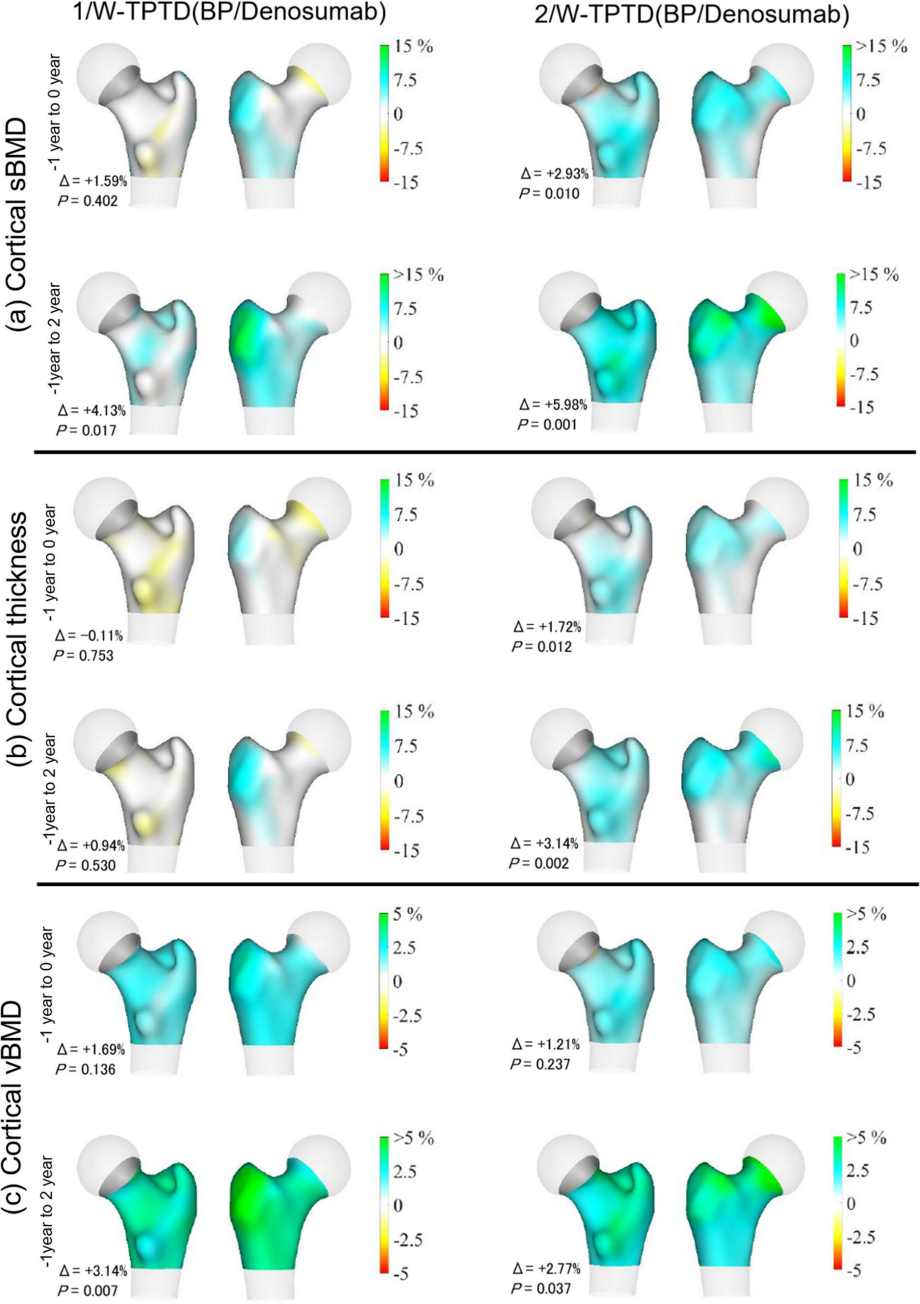
3D visualizations showing percentage changes from the start of pre-treatment to 2 years after starting post-treatment in cortical sBMD (**a**), cortical thickness (**b**), and cortical vBMD (**c**). − 1 year to 0 year, from pre-treatment to the start of post-treatment; − 1 year to 2 years, from pre-treatment to 2 years after starting post-treatment. There were 15 cases in each group. *BMD* bone mineral density; *1/W TPTD* once-weekly teriparatide; *2/W TPTD* twice-weekly teriparatide.

In the 1/W-TPTD (BP/denosumab) and 2/W-TPTD (BP/denosumab) groups, significant increases in cortical sBMD and cortical vBMD were observed at 2 years after starting post-treatment (Fig. 4). Moreover, the cross sections showing changes in cortical and trabecular vBMD at 1 year and 3 years are indicated in Supplement 8.

The analysis results when distinguishing between BP and denosumab are shown in Supplement 9. When distinguished, the same trends were observed 2 years after the start of sequential administration.

## Discussion

The study results suggest that changes in BMD values in the femoral bone and lumbar spine may vary depending on the difference in post-treatment after the end of 1/W-TPTD or 2/W-TPTD administration. Particularly for 2/W-TPTD, it was shown that maintenance therapy with BP or denosumab can further increase BMD. Furthermore, 2D-DXA images were re-evaluated by 3D modeling. Examination of morphological changes in the trabecular and cortical bones showed that structures of the trabecular bones in the neck and trochanter were improved particularly in the group 2/W-TPTD, and the cortical parameters were also improved by post-treatment of BP or denosumab.

### Two-year changes in BMD by post-treatment in the groups 1/W-TPTD and 2/W-TPTD

Valid results could be obtained for changes in BMD at a year after starting post-treatment. As for sequential administration after 1/W-TPTD, Sugimoto et al. and Mori et al. reported that BMD was maintained by BP after 72-week TPTD administration [10, 17]. Based on the report by Sugimoto et al., when BP was administered over a year after 72-week 1/W-TPTD, percentage changes in BMD from the start of 1/W-TPTD were + 9.6% in the lumbar spine and + 2.9% in the neck. In our present survey, percentage changes in BMD from the start of 1/W-TPTD to a year after starting post-treatment in the 1/W-TPTD (BP/denosumab) group were + 9.3% in the lumbar spine and −0.2% in the neck (Supplement 4). Despite the difference in the duration of 1/W-TPTD, the percentage changes in the lumbar spine were close, which supported the benefit of the sequential administration of BP/denosumab. In contrast, neck BMD did not change obviously with sequential therapy from 1/W TPTD to BP/denosumab. Nevertheless, the percentage change in BMD from the start of 2/W-TPTD to a year after starting post-treatment was + 4.9% in the group 2/W-TPTD, which was slightly higher than the value reported by Sugimoto et al. In our survey, since the duration of TPTD administration was 48 weeks and the drug for sequential administration was BP/denosumab, a closer comparison was not available. On the other hand, the increase rate of neck BMD at the end of 1/W-TPTD in the population in the present study was slightly lower than the overall results of the clinical trial [14], indicating that it might be difficult to obtain relatively favorable results of neck BMD for the population.

As for changes in BMD at 2 years after starting post-treatment, Leder et al. also reported that the percentage change in BMD of the lumbar spine from the time of drug switching to 2 years after switching was + 8.6% in a study of 2-year DAILY TPTD administration followed by switching to denosumab [18]. In our study, the percentage changes in BMD were + 8.4% in the group 1/W-TPTD (BP/denosumab) and + 6.0% in the group 2/W-TPTD (BP/denosumab) (Fig. 2). Given that the percentage changes in BMD from the start of TPTD were slightly higher in the group 2/W-TPTD (BP/denosumab), those results might be appropriate despite variations in the percentage changes depending on the starting time point. In the lumbar spine, changes in BMD over 2 years after the start of post-treatment (BP/denosumab) in our study were suggested not to significantly differ from the values in cases of 2-year DAILY TPTD followed by sequential administration of denosumab. Based on a report, sequential administration from D-TPTD to denosumab significantly increased BMD in the lumbar spine compared to monotherapy with denosumab [19]; TPTD may potentially enhance the effectiveness of other therapeutic agents administered following its use. The number of reports on the benefits of sequential administration from 1/W-TPTD or 2/W-TPTD is limited, and further studies are warranted to provide additional data.

### Changes in parameters of 3D-SHAPER from the start of 1/W-TPTD or 2/W-TPTD to 2 years after starting post-treatment

A previous comparison study between denosumab and DAILY TPTD reported contrasting results as follows: changes in cortical vBMD at 24 months after starting administration of denosumab and DAILY TPTD were + 14.9 mg/cm^3^ and − 8.0 mg/cm^3^, respectively [20].

Moreover, in another comparison study between abaloparatide and DAILY TPTD, abaloparatide showed a significant increase in cortical vBMD by 1.3% at 18 months, while the percentage change by DAILY TPTD was 0.4% without significance. As for cortical vBMD at a year after starting pre-treatment and 2 years after starting post-treatment in our study, 1/W-TPTD (BP/denosumab) significantly increased all neck, trochanter, shaft, and total hip values by 3.1% or higher, while 2/W-TPTD(BP/denosumab) also significantly increased all neck, trochanter, shaft, and total hip values by 2.5% or higher. In previous reports, the effects of TPTD preparations on cortical vBMD might seem negative. However, our present report suggests that 1/W-TPTD and 2/W-TPTD may significantly increase cortical vBMD by adequate sequential administration.

DAILY TPTD has been reported to increase trabecular vBMD in the proximal femur, decrease cortical vBMD, and significantly increase overall vBMD and bone strength parameters by continuous treatment with denosumab after the end of DAILY TPTD [21]. However, there are few reports on the effects of 1/W-TPTD and 2/W-TPTD on vBMD.

Our study results are opposite to those of previous reports that showed a decrease in cortical vBMD by the treatments, suggesting that the effects of 1/W-TPTD and 2/W-TPTD on cortical vBMD may differ from DAILY TPTD. In a clinical study for the evaluation of the radius and tibia by Chiba et al., trabecular volumetric BMD (Tb.vBMD) was significantly improved by DAILY TPTD compared to 1/W-TPTD, while cortical volumetric tissue mineral density (Ct.vTMD) was maintained by 1/W-TPTD and decreased by DAILY TPTD [22]. The effects of TPTD on the cortical bone structure may vary depending on its administration method. Zebaze et al. reported that daily administration of high-dose TPTD increased cortical porosity in rabbits, while its weekly administration did not increase cortical porosity even at a higher dose [23]. Our study also showed that 1/W-TPTD and 2/W-TPTD increased rather than decreased cortical vBMD. Such results are supported by Tsukamoto et al., who performed a study to examine bone microstructure with HR-pQCT at 48 weeks after 1/W-TPTD and 2/W-TPTD and reported that mean changes in cortical porosity were only 0.3% and 0.2%, respectively, in the tibia and radius [24]. Cortical porosity and decrease in cortical vBMD due to TPTD had previously been implicated in causing a decrease in cortical strength, which can be dispelled by the results of our present study. We also reported trends toward more favorable improvement of the cortical bone parameters by 1/W-TPTD and 2/W-TPTD [25]. The results of this study provide evidence for sequential administration, providing further support.

Based on the present study results, BMD and 3D-SHAPER indicators were improved by 2/W-TPTD compared to 1/W-TPTD; the cause is unknown, which may be a subject to be examined in the future.

There were some limitations in the study. Data collection was limited to subjects for whom DXA measurement device for analyses with the same software was used. Thus, a bias could not be excluded for patient selection. Furthermore, since this is less than 10% of the original number of participants, it is possible that the results cannot be generalized. Since data in actual clinical settings were collected retrospectively, the number of subjects from whom DXA data could be collected in the group 2/W-TPTD (others) was limited to five, and the sample size could not be calculated for subgroup analysis of the post-treatment. In a particular group with a small sample size, the desire is to accumulate more subjects for analyses. Furthermore, there were only five male participants. The results excluding men showed approximately the same trend (data not shown), but more cases would be needed to verify the effects on men. This study is a retrospective analysis of routine insurance-covered medical care administered after the completion of the TWICE trial, and the anti-resorptive agents used were determined between the attending physician and the patient. Thus, the point represents a limitation of this study, and we need further investigations.

### Conclusion

We retrospectively conducted a follow-up study after the completion of the multicenter, randomized, double-blind, double-dummy, active-controlled, non-inferiority trial (TWICE study) to evaluate the effects of sequential therapy after 2/W-TPTD or 1/W-TPTD. In subjects who received 1/W-TPTD and 2/W-TPTD over about 1 year followed by sequential administration of BP or denosumab, significant improvement of BMD was continuously observed. Furthermore, significant improvement of cortical vBMD was also shown by analysis using 3D-SHAPER. 1/W-TPTD and 2/W-TPTD were suggested to become more effective for treatment of osteoporosis by appropriate selection of a drug for sequential administration.

## Supplementary Information

Below is the link to the electronic supplementary material.

Supplementary file1 (PDF 91 KB)

Supplementary file2 (PDF 170 KB)

Supplementary file3 (TIFF 435 KB)

Supplementary file4 (TIF 202 KB)

Supplementary file5 (TIF 65 KB)

Supplementary file6 (TIF 113 KB)

Supplementary file7 (PDF 281 KB)

Supplementary file8 (PDF 271 KB)

Supplementary file9 (PDF 55 KB)

## Data Availability

The biochemical data used to support the findings of this study are available from the corresponding author upon request.
