# Supplementary figures and images for: The effects of sequential therapy using anti-resorptive agents after administering once-weekly teriparatide or twice-weekly teriparatide
Source: J Bone Miner Metab. 2026 Jan 31;44(3):363–74. doi: 10.1007/s00774-026-01690-7 (PMC13246890; doi:10.1007/s00774-026-01690-7)

Supp.1  
Handling sequential administration

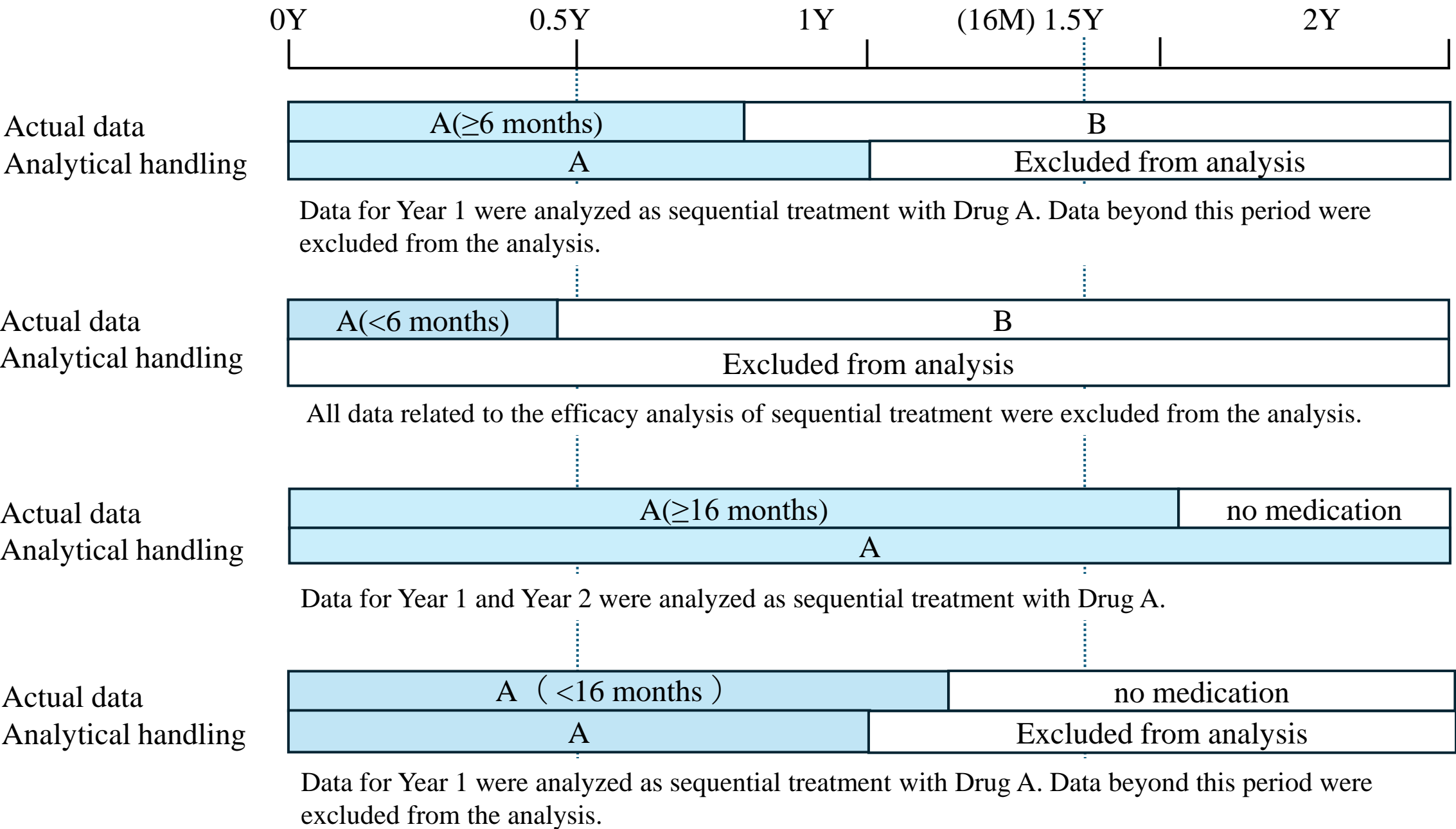

Supplement: Supplementary file 1 — Supplementary file1 (PDF 91 KB) [file 774_2026_1690_MOESM1_ESM.pdf]

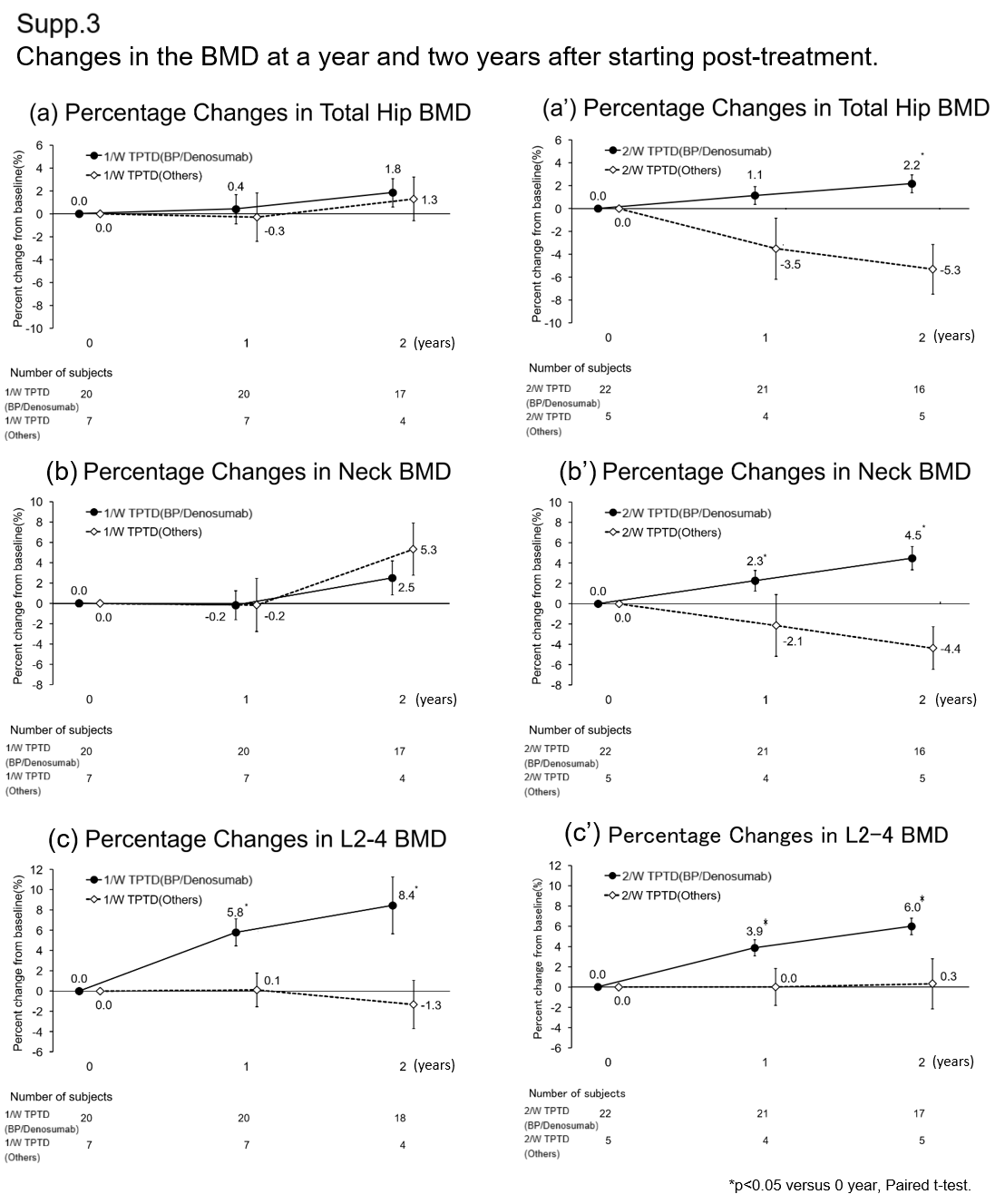

Supplement: Supplementary file 3 — Supplementary file3 (TIFF 435 KB) [file 774_2026_1690_MOESM3_ESM.tiff]

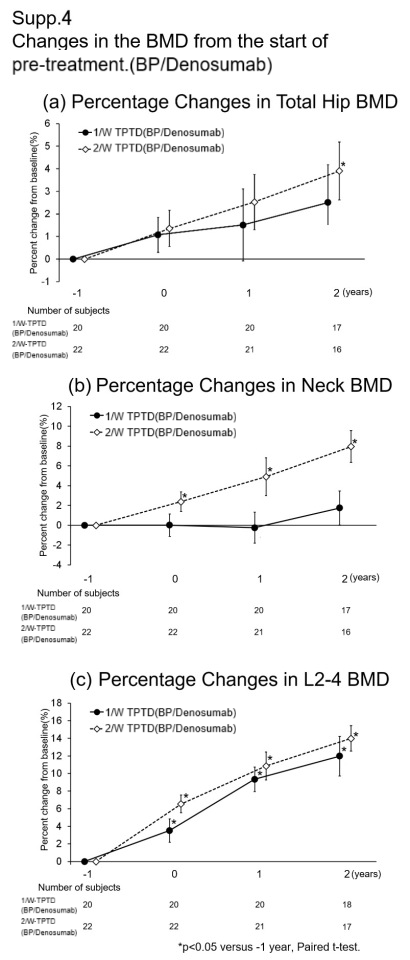

Supplement: Supplementary file 4 — Supplementary file4 (TIF 202 KB) [file 774_2026_1690_MOESM4_ESM.tif]

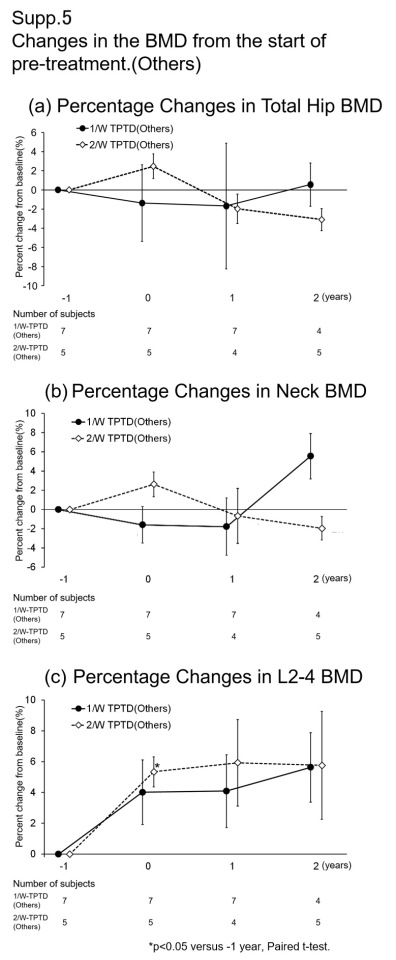

Supplement: Supplementary file 5 — Supplementary file5 (TIF 65 KB) [file 774_2026_1690_MOESM5_ESM.tif]

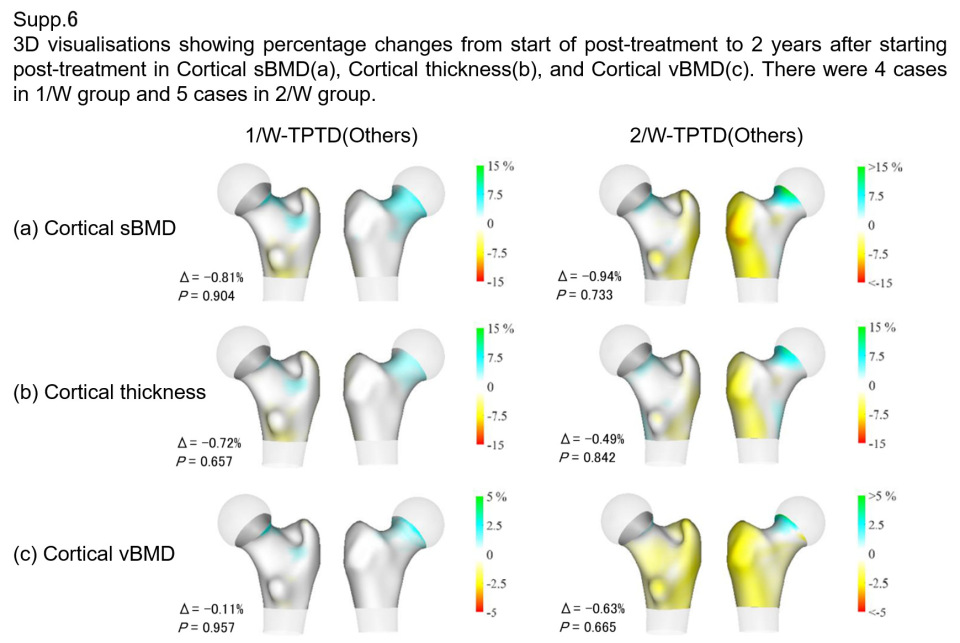

Supplement: Supplementary file 6 — Supplementary file6 (TIF 113 KB) [file 774_2026_1690_MOESM6_ESM.tif]
