# Supplementary material for: The effects of sequential therapy using anti-resorptive agents after administering once-weekly teriparatide or twice-weekly teriparatide
Source: J Bone Miner Metab. 2026 Jan 31;44(3):363–74. doi: 10.1007/s00774-026-01690-7 (PMC13246890; doi:10.1007/s00774-026-01690-7)
Supplement: Supplementary file 2 — Supplementary file2 (PDF 170 KB) [file 774_2026_1690_MOESM2_ESM.pdf]

Supplement 2

	BP/Denosumab	Others	p	
n	42	12		
Age (years)	74.5 ± 0.9	76.7 ± 1.5	0.243	a
Heigh (cm)	150 ± 1.0	151.1 ± 1.7	0.609	a
Weight (kg)	50.7 ± 1.0	53.9 ± 2.2	0.157	a
Body Mass Index (kg/m^2)	22.7 ± 0.6	23.7 ± 1.2	0.408	a
25-OHvitaminD3 (ng/mL)	25.8 ± 1.1	25.8 ± 1.9	0.993	a
Estimated GFR ($mL/min/1.73m^2$)	65.5 ± 2.2	65.3 ± 4.2	0.954	a
Complications				
Type 2 diabetes mellitus	2 (4.8%)	1 (8.3%)	0.537	b
Chronic kidney disease (CKD)	3 (7.1%)	1 (8.3%)	1.000	b
Hypertension	6 (14.3%)	3 (25.0%)	0.399	b
Dyslipidemia	4 (9.5%)	2 (16.7%)	0.605	b
Knee osteoarthritis	3 (7.1%)	4 (33.3%)	0.036	b
Sex				
Male	5 (11.9%)	0 (0.0%)	0.575	a
Female	37 (88.1%)	12 (100.0%)		
Postmenopausal duration (years)				
10 - <20	10 (23.8%)	0 (0.0%)	0.049	b
≥20	27 (64.3%)	12 (100.0%)		
Male	5 (11.9%)	0 (0.0%)		
Non-vertebral fractures without large external force occurring at or after age 50 years				
Yes	15 (35.7%)	4 (33.3%)	1.000	b
No	27 (64.3%)	8 (66.7%)		
Medical history relevant to bone metabolism				
Yes	5 (11.9%)	2 (16.7%)	0.645	b
No	37 (88.1%)	10 (83.3%)		
Current smoking				
Yes	1 (2.4%)	0 (0.0%)	1.000	b
No	41 (97.6%)	12 (100.0%)		
Alcohol consumption (3 or more units/day)				
Yes	0 (0.0%)	0 (0.0%)	NA	b
No	42 (100.0%)	12 (100.0%)		
Parent fractured hip				
Yes	8 (19.0%)	1 (8.3%)	0.665	b
No	34 (81.0%)	11 (91.7%)		
Prior medications for osteoporosis				

Yes	22 (52.4%)	8 (66.7%)	0.515	b
No	20 (47.6%)	4 (33.3%)		
Number of vertebral fractures at baseline				
0	10 (23.8%)	4 (33.3%)	0.922	b
1	24 (57.1%)	6 (50.0%)		
2 to3	7 (16.7%)	2 (16.7%)		
4 to 5	0 (0.0%)	0 (0.0%)		
Missing or not reported	1 (2.4%)	0 (0.0%)		
Lumbar spine BMD (based on YAM) (L2-L4) (%)	67 ± 1.4	70.5 ± 2.3	0.216	a
Lumbar spine BMD (based on YAM) (L1-L4) (%)	0.7 ± 0	0.7 ± 0	0.248	a
Femoral neck BMD (based on YAM) (%)	65.5 ± 1.6	67.8 ± 2.5	0.480	a
Total hip BMD (based on YAM) (%)	72.6 ± 1.5	78.7 ± 2.4	0.054	a

a: Student's t-test; b: Fisher’s exact test.
