# Supplementary material for: The effects of sequential therapy using anti-resorptive agents after administering once-weekly teriparatide or twice-weekly teriparatide
Source: J Bone Miner Metab. 2026 Jan 31;44(3):363–74. doi: 10.1007/s00774-026-01690-7 (PMC13246890; doi:10.1007/s00774-026-01690-7)

Supp.7

Cross-sections showing changes in cortical and trabecular vBMD at 2 years after starting sequential administration

1/W-TPTD(BP/Denosumab)

Mid-coronal

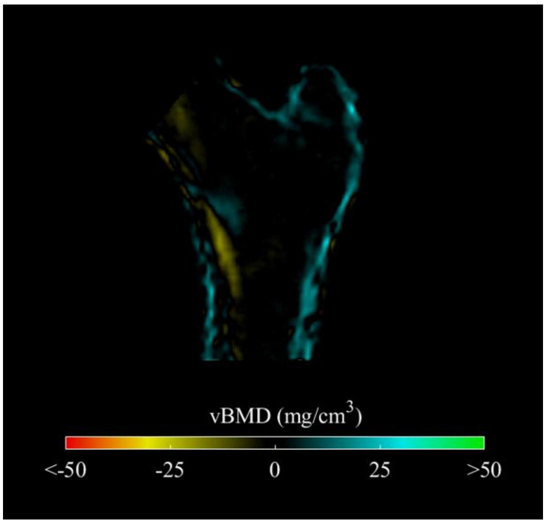

Neck

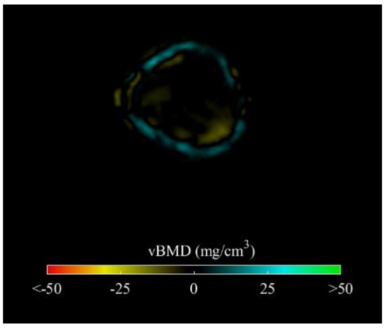

Intertrochanteric

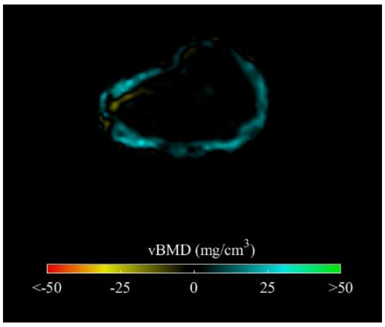

Lower shaft

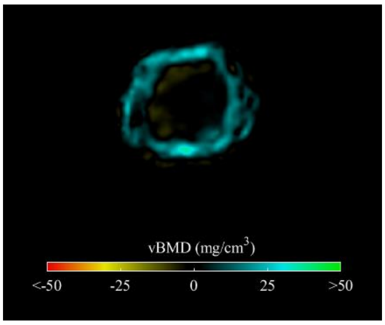

1/W-TPTD(others)

Mid-coronal

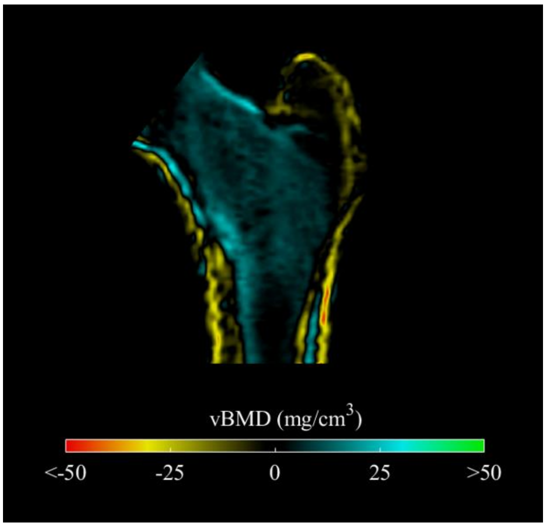

Neck

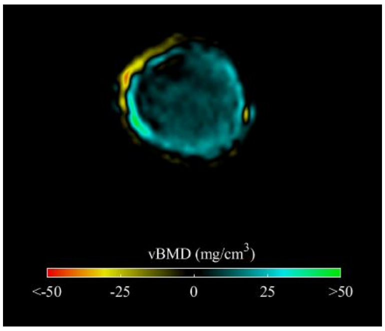

Intertrochanteric

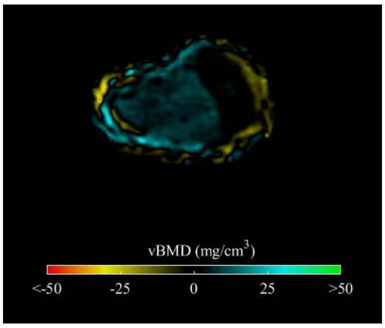

Lower shaft

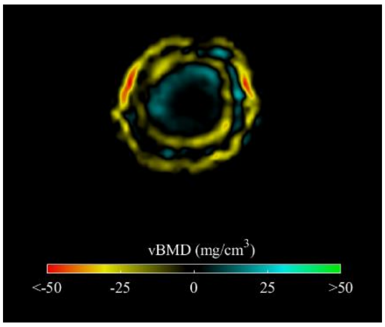

Cross-sections showing changes in cortical and trabecular vBMD at 2 years after starting sequential administration

2/W-TPTD(BP/Denosumab)

Mid-coronal

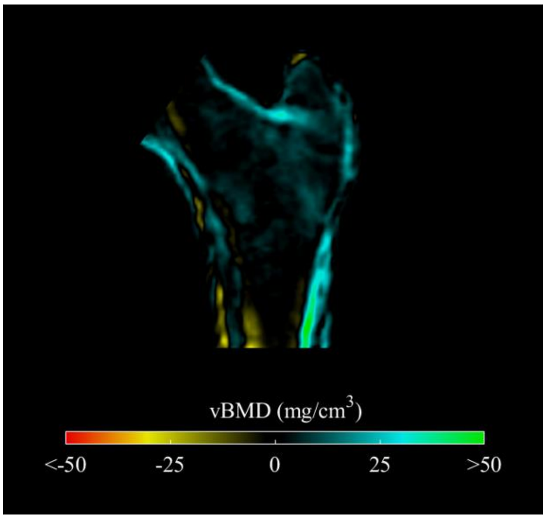

Neck

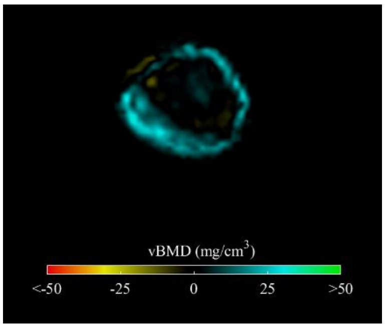

Intertrochanteric

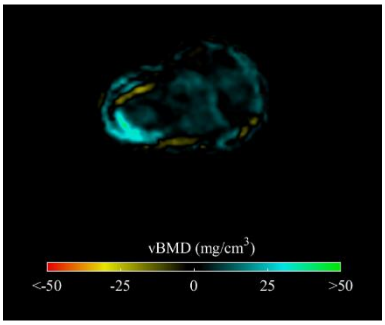

Lower shaft

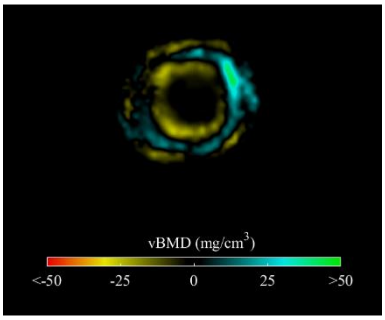

2/W-TPTD(others)

Mid-coronal

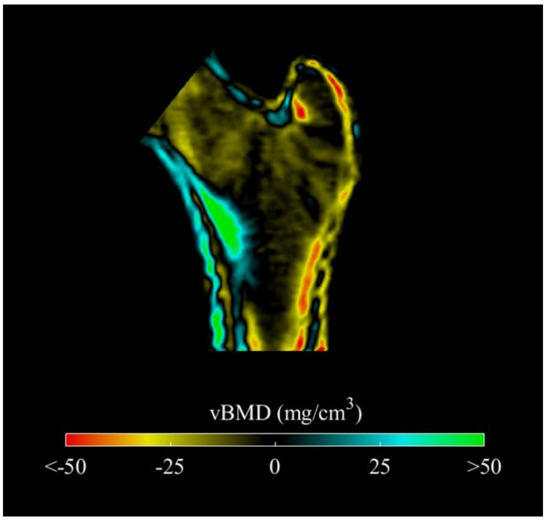

Neck

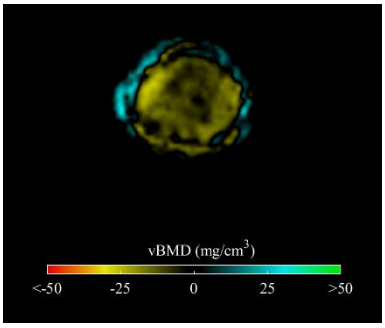

Intertrochanteric

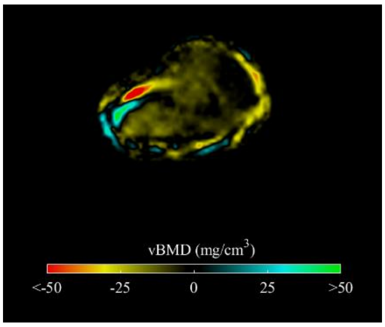

Lower shaft

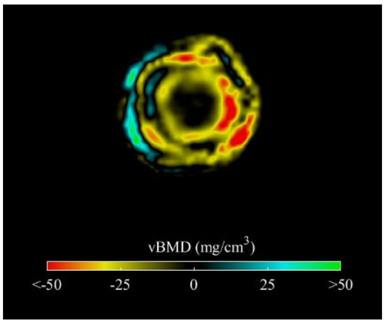

Supplement: Supplementary file 7 — Supplementary file7 (PDF 281 KB) [file 774_2026_1690_MOESM7_ESM.pdf]
