# Supplementary material for: The effects of sequential therapy using anti-resorptive agents after administering once-weekly teriparatide or twice-weekly teriparatide
Source: J Bone Miner Metab. 2026 Jan 31;44(3):363–74. doi: 10.1007/s00774-026-01690-7 (PMC13246890; doi:10.1007/s00774-026-01690-7)

Supp.8

Cross-sections showing changes in cortical and trabecular vBMD (-1year to 0year)

1/W-TPTD(BP/Denosumab)

Mid-coronal

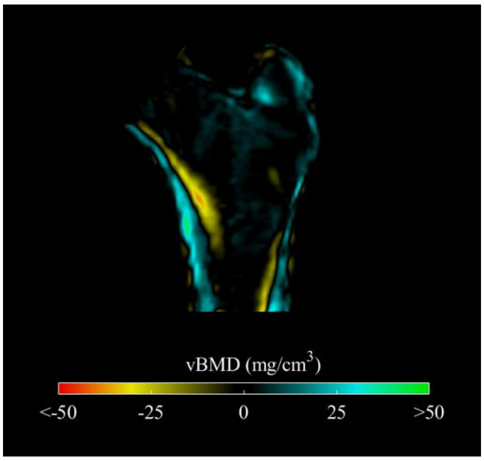

Neck

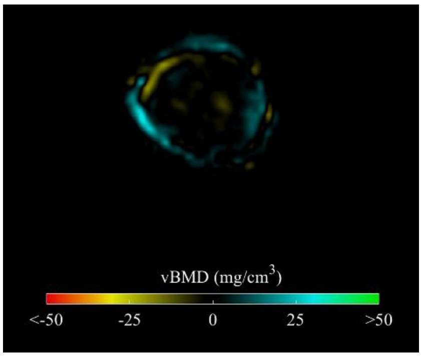

Intertrochanteric

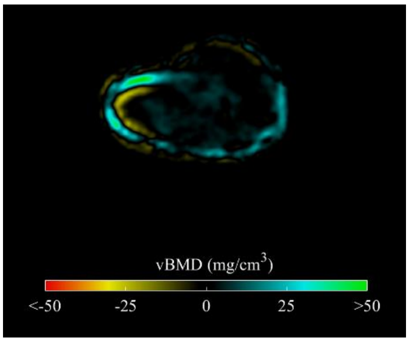

Lower shaft

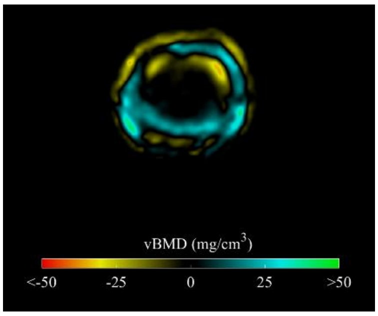

2/W-TPTD(BP/Denosumab)

Mid-coronal

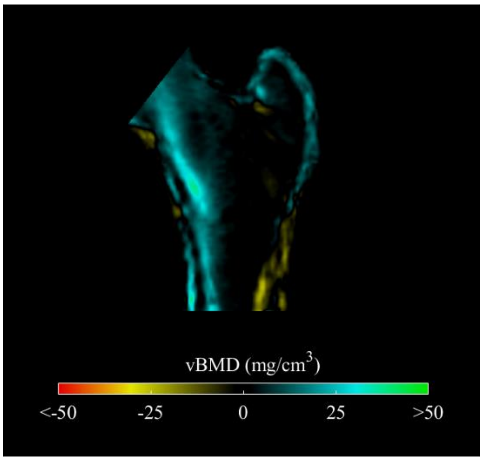

Neck

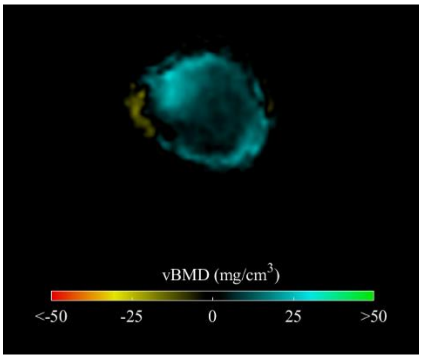

Intertrochanteric

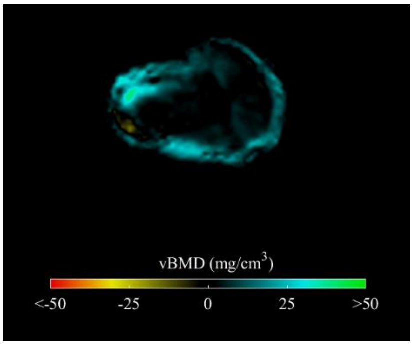

Lower shaft

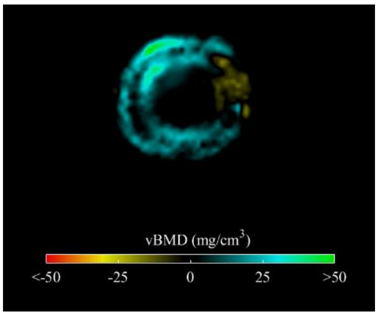

Cross-sections showing changes in cortical and trabecular vBMD (-1year to 2years)

1/W-TPTD(BP/Denosumab)
Mid-coronal

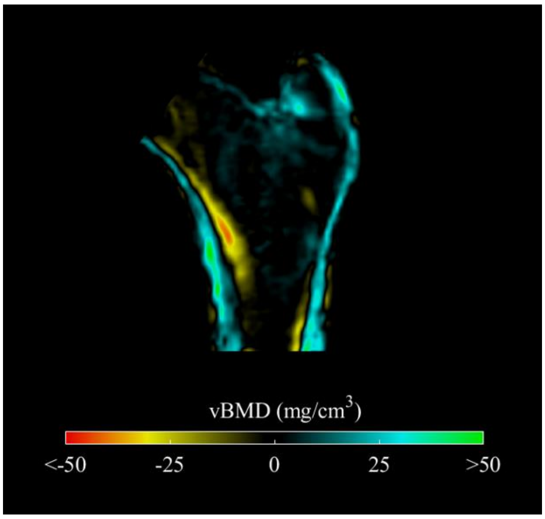

2/W-TPTD(BP/Denosumab)
Mid-coronal

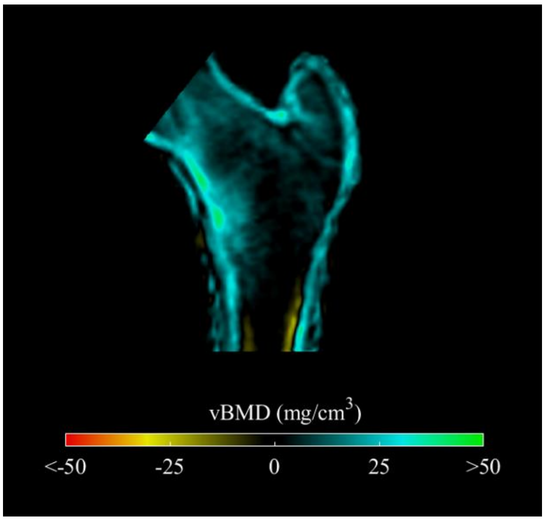

Neck

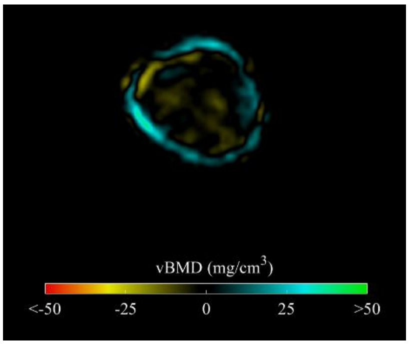

Neck

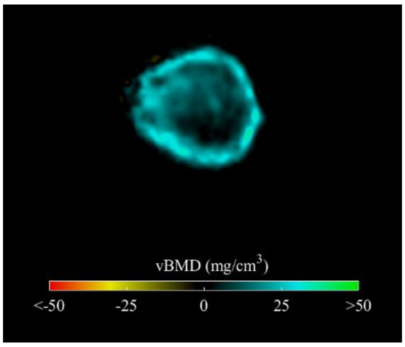

Intertrochanteric

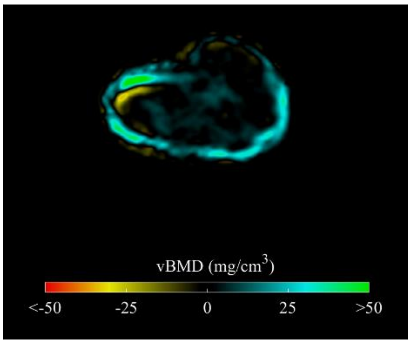

Intertrochanteric

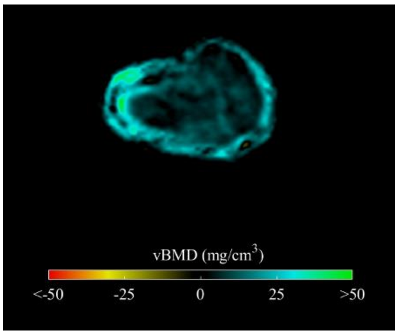

Lower shaft

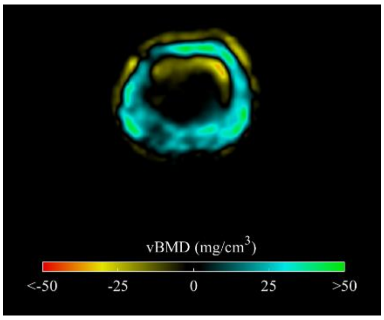

Lower shaft

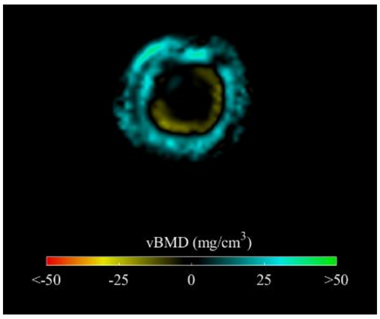

Supplement: Supplementary file 8 — Supplementary file8 (PDF 271 KB) [file 774_2026_1690_MOESM8_ESM.pdf]
