# Supplementary material for: The effects of sequential therapy using anti-resorptive agents after administering once-weekly teriparatide or twice-weekly teriparatide
Source: J Bone Miner Metab. 2026 Jan 31;44(3):363–74. doi: 10.1007/s00774-026-01690-7 (PMC13246890; doi:10.1007/s00774-026-01690-7)
Supplement: Supplementary file 9 — Supplementary file9 (PDF 55 KB) [file 774_2026_1690_MOESM9_ESM.pdf]

Supp. 9

Changes in the BMD and femoral bone parameters of 3D-SHAPER at a year and two years after starting post-treatment

		1/W-TPTD BP					2/W-TPTD BP					1/W-TPTD Denosumab					2/W-TPTD Denosumab				
		n	mean	SE	p	*	n	mean	SE	p	*	n	mean	SE	p	*	n	mean	SE	p	*
Total Hip BMD	0Y to 1Y(%)	14	0.736	1.671	0.710		16	0.373	0.690	0.625		6	-0.367	1.822	0.901		5	3.584	2.207	0.193	
	0Y to 2Y(%)	11	1.723	1.811	0.421		11	1.269	0.880	0.188		6	2.039	1.334	0.177		5	4.163	1.209	0.021	*
Neck BMD	0Y to 1Y(%)	14	-0.150	1.917	0.718		16	1.508	1.097	0.185		6	-0.227	1.745	0.921		5	4.706	2.423	0.128	
	0Y to 2Y(%)	11	1.558	2.442	0.666		11	5.118	1.442	0.004	*	6	4.200	1.646	0.057		5	3.150	1.874	0.177	
L2-4 BMD	0Y to 1Y(%)	14	5.401	1.736	0.007	*	16	3.021	0.875	0.003	*	6	6.655	1.923	0.013	*	5	6.540	1.259	0.010	*
	0Y to 2Y(%)	12	8.798	4.197	0.048	*	12	5.214	0.975	<0.001	*	6	7.743	1.503	0.003	*	5	7.822	1.244	0.008	*
Cortical sBMD	0Y to 2Y(%)	9	0.811	1.369	0.780		10	2.297	1.720	0.215		6	5.210	1.897	0.033	*	5	4.468	1.838	0.037	*
Cortical vBMD	0Y to 2Y(%)	9	-0.630	1.303	0.518		10	1.063	1.046	0.350		6	4.752	1.219	0.012	*	5	2.558	1.377	0.140	
Cotical thickness	0Y to 2Y(%)	9	1.474	0.854	0.139		10	1.183	0.965	0.245		6	0.425	1.209	0.759		5	1.860	1.067	0.149	

*p<0.05 versus 0-year, Paired t-test.

Changes in BMD and femoral bone parameters of 3D-SHAPER from the start of pre-treatment to two years after starting post-treatment

		1/W-TPTD BP					2/W-TPTD BP					1/W-TPTD Denosumab					2/W-TPTD Denosumab				
		n	mean	SE	p	*	n	mean	SE	p	*	n	mean	SE	p	*	n	mean	SE	p	*
Total Hip BMD	-1Y to 0Y(%)	14	1.398	1.016	0.219		17	1.153	0.993	0.316		6	0.321	1.098	0.834		5	2.060	1.100	0.142	
	-1Y to 1Y(%)	14	2.152	2.013	0.362		16	1.513	1.208	0.286		6	0.013	2.620	1.000		5	5.786	3.184	0.138	
	-1Y to 2Y(%)	11	2.554	2.382	0.372		11	2.791	1.541	0.111		6	2.411	2.186	0.327		5	6.342	2.145	0.034	*
Neck BMD	-1Y to 0Y(%)	14	-0.057	1.426	0.917		17	1.658	1.038	0.183		6	0.161	1.880	0.940		5	4.852	2.609	0.121	
	-1Y to 1Y(%)	14	-0.298	2.083	0.684		16	3.315	1.817	0.090		6	-0.119	2.111	0.984		5	10.013	5.282	0.116	
	-1Y to 2Y(%)	11	0.345	2.374	0.982		11	7.824	1.565	<0.001	*	6	4.313	2.073	0.084		5	8.254	4.143	0.101	
L2-4 BMD	-1Y to 0Y(%)	14	3.675	1.371	0.018	*	17	6.299	1.278	<0.001	*	6	3.150	3.223	0.417		5	7.349	1.071	0.001	*
	-1Y to 1Y(%)	14	9.131	1.697	<0.001	*	16	9.733	1.939	<0.001	*	6	9.827	2.752	0.012	*	5	14.379	1.915	0.003	*
	-1Y to 2Y(%)	12	12.452	3.046	0.002	*	12	13.240	1.892	<0.001	*	6	11.029	3.162	0.013	*	5	15.757	1.925	0.002	*
Cortical sBMD	-1Y to 0Y(%)	9	1.830	5.490	0.064		10	2.362	1.342	0.110		6	-1.771	1.671	0.260		5	4.056	1.053	0.009	*
	-1Y to 2Y(%)	9	2.635	7.906	0.113		10	4.587	1.426	0.022	*	6	3.224	1.227	0.052		5	8.763	2.856	0.014	*
Cortical vBMD	-1Y to 0Y(%)	9	1.160	3.479	0.018	*	10	0.799	1.320	0.601		6	-0.817	1.270	0.495		5	2.020	0.698	0.034	*
	-1Y to 2Y(%)	9	1.287	3.860	0.079		10	1.832	1.401	0.279		6	3.870	1.476	0.062		5	4.640	1.764	0.036	*
Cotical thickness	-1Y to 0Y(%)	9	1.162	3.487	0.842		10	1.575	0.768	0.071		6	-0.927	1.606	0.548		5	2.004	1.004	0.119	
	-1Y to 2Y(%)	9	1.516	4.547	0.277		10	2.773	1.218	0.040	*	6	-0.533	1.724	0.728		5	3.883	1.131	0.021	*

*p<0.05 versus -1-year, Paired t-test.
